# Physical and Cognitive Functioning of Institutionalized Elderly People in Rural Areas. Preventive Actions Using Physical Activity and Music Therapy

**DOI:** 10.3390/healthcare9111536

**Published:** 2021-11-10

**Authors:** María José González-Ojea, Sara Domínguez-Lloria, Iago Portela-Pino, Myriam Alvariñas-Villaverde

**Affiliations:** 1 Department of Didactics, School Organization and Research Methods, Faculty of Education and Sport Sciences, University of Vigo, 36310 Pontevedra, Spain; mariajosegonzalezojea@uvigo.es; 2 Research Group on Education, Physical Activity and Health (GIES10), Galicia Sur Research Institute (IIS Galicia Sur), SERGAS-UVIGO, 36312 Vigo, Spain; iago.portela@ui.es (I.P.-P.); myalva@uvigo.es (M.A.-V.); 3 Department of Special Didactics, Faculty of Education and Sport Sciences, University of Vigo, 36310 Pontevedra, Spain; 4 Department of Health Sciences, Faculty of Health Sciences, Isabel I University, 09003 Burgos, Spain

**Keywords:** healthcare, physical activity, music therapy, alternative therapies

## Abstract

Background: Comprehensive geriatric evaluation should include a functional and cognitive assessment to guide the intervention of interdisciplinary teams. The aim of this study was to analyze the physical capacities of institutionalized elderly people and to describe the preventive actions of physical activity and music therapy as non-invasive preventive pharmacological treatments given their importance for the cognitive and functional performance of elderly people. An observational and descriptive cross-sectional study was carried out. The participants in the study were 109 elderly people institutionalized in three residential centers with a mean age of 83.41 years (SD = 8.72). Findings: Most of the residents had very impaired physical faculties. However, cognitive impairment was not very high. Most residents (55.04%) had some form of dementia and/or high blood pressure (54.12%) followed by pathologies such as diabetes (27.52%), heart failure (17.43%), Parkinson’s disease (9.17%) and chronic obstructive pulmonary disease (8.25%). There were no differences in cognitive or physical capacity among the residents according to sex, age, or education and only those who had worked in the service sector had less cognitive capacity than those who had worked in the agricultural sector or as housewives. Applications: Facilitate the creation and development of programs based on physical activity and music therapy in residential centers that can prevent and improve pathologies on the elderly.

## 1. Introduction

### 1.1. Functional and Cognitive Situation of the Elderly

Every year, the global burden of disease (GBD) study measures the health of the world population. In the list of causes of the GBD, three categories are included: transmissible diseases, maternal, neonatal and nutritional; non-communicable diseases (NCDs) and accidents.

The largest cause of death in the elderly is NCDs. Globally this is related to physical and psychological variables [1,2,3].

Martín and Barros [4] show that 73.4% (95% uncertainty interval [UI] 72.5–74.1) of the total deaths in 2017 were the result of NCDs, while communicable, maternal, neonatal, and nutritional (CMNN) causes accounted for 18.6% (17.9–19.6) and accidents for 8.0% (7.7–8.2). Aging makes elderly people prone to suffer different diseases at the same time. In fact, a characteristic of older people is the possibility of suffering from comorbidity.

Regarding the health of institutionalized older people, according to a study by Maestre-Miquel et al. [5] 66.7% present dependence, the percentage being higher among women. Among women older than 85 years, 88.6% had comorbidity, while 79.8% of men of the same age group had comorbidity. It is noteworthy that only 6.6% of patients with comorbidity received healthy recommendations during the consultation. These authors also found a lack of preventive action and health promotion among the elderly, with differences between hospitals and geriatric residences. Therefore, it seems necessary to encourage a health-promoting attitude and preventive interventions in gerontological clinical practice.

Other studies, like that of Rodríguez Díaz, et al., [6], also find a relationship between the degree of emotional distress and functional dependence in people over 60 years of age. In fact, people with greater anxious and depressive symptoms are those who present greater physical dependence. In general, the loss of active roles, dependence and decreased quality of life increase depressive symptoms [7].

Also, aging makes them prone to different diseases at the same time. In fact, a characteristic of older people is the possibility of suffering from comorbidity [8].

In recent years Alzheimer’s disease has been the most common form of dementia, accounting for 60% of the cases. It is an incurable disease characterized by cognitive, non-cognitive and functional deficits [9]. Dementia causes high disability among the elderly [10]. With increasing life expectancy in most countries, it has been estimated that the prevalence of dementia will continue to grow significantly over the next two decades. Dementia leads to cognitive impairments, particularly short-term memory loss and impairments in functioning and quality of life [11].

For the prevention of these disabilities/pathologies there is a series of alternative therapies that can favor improvement while delaying the deterioration of institutionalized elderly people, such as moderate physical activity or music therapy.

### 1.2. Benefits of Physical Activity and Music Therapy in the Elderly

Many studies have found positive effects for the practice of physical activity during old age. Among them are a reduction of the level of depression [12] a tool to fight against cognitive decline, improve memory and delay the onset of dementia [13]. It also provides bodily, emotional, and social improvements [14] or helps in reducing frailty [15] and preventing falls with a consequent increase in quality of life.

The use of music therapy as an alternative therapy focused on the decline of balance in the elderly using progressive rhythm [16] is well known. Such activities do not only have a physical impact, but they also foster improvements in interpersonal relations, communication, and other relevant physio-therapeutic aspects. The use of music therapy accompanied by physical exercise is a reliable therapeutic tool in rehabilitation programs, decreasing the risk of associated diseases and activating a neuro protector effect [17].

The aim is to work on rhythm through music therapy. To be truly effective, specific tasks must be encouraged such as: spin, weight on one foot, exercises on unstable surfaces, exercises with different weight loads so improving balance and walking. That way, the elderly person indirectly improves their mood state because rhythm and music offer a distraction from feelings of fatigue or fear of falling [18].

Currently, many people do not reach the basic physical requirements allowing them functional independence and far from improving, these rates are worsening [19]. Day and geriatric centers offer a great opportunity for this population to engage in daily physical activity through occupational therapy. However, most facilities do not have a specific program focused on the practice of physical exercise [20].

Therefore, the aim of this study is to analyze the sociodemographic profile and the physical capabilities of institutionalized elderly people and to describe the educational applications of physical activity and music therapy as non-invasive preventive pharmacological treatments given the importance of paying attention to the cognitive and functional performance of the elderly in three geriatric residences in a rural environment.

## 2. Materials and Methods

This article is part of a more extensive investigation where the data described here refers to the diagnostic evaluation performed.

Ethical research protocols were followed, with a special emphasis on confidentiality.

Since it is an observational, descriptive, cross-sectional study, the deontological standards recognized by the Declaration of Helsinki (Hong Kong revision, September 1989, updated to 2013), the code of good scientific practices approved by the Higher Center for Scientific Research (CSIC) in March 2010 and the good practice agreements adopted by the Committee on Publication Ethics (COPE), as well as the AERA standards.

In a second phase of intervention, a mandatory report was requested from the Research Ethics Committee (CEI) of the GIES10 research group (Cod. 02/2019).

### 2.1. Participants

The participants were 109 elderly individuals institutionalized in rural residences located in towns of from 8000 to 20,000 citizens. Of these, 71 were women and 28 men and 50.5 % of the residents were single and 49.5% were married, with a mean age of 83.41 years (SD = 8.72).

### 2.2. Instruments

The Barthel and Pfeiffer scales were used for functional and cognitive assessment. The former measures functionality for the activities related to daily living (eating, washing, dressing, grooming, bowel movements, urination, toileting, ambulation, transferring and stairs) and thus allows determination of the degree of dependence of a person and, therefore, the need for assistance in the 10 activities mentioned [21].

The Pfeiffer scale (Short Portable Mental Status Questionnaire) is a very brief questionnaire of only 10 items, but with high sensitivity and specificity indexes measuring the functions of orientation, recall memory, concentration, and calculation [22].

In the Spanish versions, the Cronbach’s alpha coefficient of both scales is always greater than 0.070. [23,24,25].

To information on diagnoses, the medical records of the patients were reviewed, and a key was given to classify them.

### 2.3. Procedure

The three existing geriatric residences in the same region or administrative unit were selected and all were in rural areas. The questionnaire was administered individually and in person to institutionalized patients by health personnel. After communicating the appropriate instructions and with prior informed consent, all the residents voluntarily completed the information requested. Those patients who gave their consent and could answer the questions asked in both instruments were selected.

### 2.4. Data Analysis

For data analysis, the statistical program SPSS 21.0 (IBM, Armonk, NY, USA, 2013) was used.

A descriptive analysis of the items, mean, standard deviation and confidence interval was carried out, as well as the calculation of the Student’s *t* for the dichotomous independent variables and ANOVA with the polytomous independent variables. The Kolmogorov–Smirnov test was used in order to decide in a rigorous way whether or not the available sample came from a normal distribution. Given that Sig = 0.405 it was concluded that the dependent variable was normally distributed and, therefore, parametric statistics were used.

## 3. Results

With respect to the health characteristics of the residents of the 109 residents assessed, 27.52% suffered from diabetes, 55.04% from some type of dementia, 9.17% from Parkinson’s disease, 17.43% from heart failure, only one person suffered from benign prostatic hyperplasia, 8.25% from chronic obstructive pulmonary disease, and 54.12% from arterial hypertension.

Regarding their physical situation and psychological characteristics, we found that most of the residents had very deteriorated physical faculties since the mean was 2.88 when the maximum score on the scale was 10 and the confidence interval was between 2.28 and 3.48. However, cognitive impairment was not very high since the mean was 65.46 when the maximum score was 100 and its confidence interval was between 64.63 and 75.97 (Table 1).

If we group the results (Table 2) we see that most of the residents, 47.7%, are slightly impaired, 11% are moderately impaired, 16.5% are totally independent and 11% are severely impaired, but 13.8% are totally dependent. If we group the results that measure memory and orientation, we find that we have not been able to assess 9.2% of the residents because they present a very profound mental impairment, 46.8% are normal, 32.1% are deficient, and 11.9% are severely impaired.

Most residents had children (56.9%). The mean number of children per resident was therefore very low at 1.38. That is, 43.1% had no children, 21.1% had only one child, 16.5% had two children and 19.3% had more than three children. These data corresponded to the percentage of unmarried individuals in the sample given that, in this age group, during their youth it was more difficult to have children outside of marriage.

Their level of education was related to the work they performed. It was low since almost 90% of the residents had only primary education. This is also explained by the fact that the program was carried out in a residence that mainly accommodates a low-income population.

With respect to the work performed by the residents in this center we found that the majority were housewives (29.4%), followed by workers in different services (25.7%), farmers (24.8%) and technicians and artisans (14.7%). Professional technicians and administrative workers (5.5%) represented smaller percentages.

It is important to note that of the total number of women in the sample (*n* = 71), 45.07% were housewives, i.e., almost half.

The rest were mainly engaged in agricultural work, which they combined with their activities at home.

There were no differences between the means according to the work they performed, except in the Pfeiffer scale, where we found differences between those who worked in the service sector and those who performed agricultural work or housework (Table 3).

As can be seen in Table 4, there were no differences between residents according to sex. Men and women presented similar means for the Pfeiffer and Barthel scales. There were also no differences with respect to whether they were parents or not in any of the scales: Pfeiffer and Barthel. With respect to level of education, there were no differences either with respect to level of dependence or with respect to memory and orientation.

## 4. Discussion and Conclusions

### 4.1. Demographic Profile of the Institutionalized Elderly

In our study, most of the participants were women, as is usual in these institutions, since the number of women over 65 years of age is greater than the number of men.

In old age, in general, women are the majority sex, something that becomes more obvious with advancing age.

In 2017, women had a life expectancy at birth of 85.7 years, and men 80.4 years, while at 65 years of age life expectancy in the female sex increased by 23 additional years and in the male sex by 19.1 additional years [26].

The level of education was very low and only 10% had more than a high school education. According to Abellán, et al. [26], the oldest people in our society are, in turn, the worst educated in academic terms. Among the elders there are groups of illiterate people or those without any kind of formal education, which can be explained by the poor living and economic conditions of their time, without schools or teachers in many cases, and which did not allow access to education for a large part of the population. These poor living conditions also influenced school absenteeism at the beginning of the 20th century, since children were forced to work [27]. In the last 40 years, the natural disappearance of part of this population and the creation of educational policies have helped to reduce illiteracy and increase the number of people with secondary or university education.

In line with the level of education of the residents and given that the study was carried out in residences located in an eminently rural region, the women were housewives and the men worked in the service sector or in agriculture. At the beginning of the 20th century, the great majority of the population in rural areas, including children, who today are our elders, and who began to work in a recognized manner at approximately 12 years of age, worked mainly in the fields, as day laborers, farmers, or cattle ranchers. Women and girls were mainly dedicated to housework, although they also helped in the fields and oversaw caring for the animals [27].

Cognitive capacity was higher in people working in the agricultural sector and housewives than in those working in the service sector.

More than half of the residents did not have children, which corresponds to the percentage of unmarried individuals in the sample. This was justified because it was those elderly without family, single and without children, who have the least resources or family support, making them, in most cases, in need of residences to be able to live accompanied in their old age and with the care that their condition requires, both cognitively and physically. At the same time, we must remember that in the period that corresponds to the youth of our elderly it was more difficult to have children out of wedlock, which justified the fact that the unmarried persons in our sample do not have children.

In fact, one of the most significant causes for entering a center, along with living alone and poor relationships with children, was the fact of not having a family [28].

### 4.2. The Health of Institutionalized Elderly People

Aging leads to the emergence of chronic pathologies and multimorbidity, which is the presence of more than one health problem [8].

As already described in the theoretical part, the pathologies suffered chronically by the elderly were multiple. Some of these pathologies, which were also present to varying degrees among the participants in this study, were arterial hypertension and dementia. In addition, we found type 2 diabetes mellitus, Parkinson’s disease, heart failure, benign prostatic hyperplasia in men and chronic obstructive pulmonary disease.

With respect to their physical and psychological situation, we found that most of the residents had very impaired physical faculties; however, cognitive impairment was not very high. There were no differences according to sex, age, whether they have children, or with respect to their level of education.

Life expectancy is increasing and, with it, so is the number of chronic diseases suffered by the elderly, and one of the main problems for them is physical deterioration, the increasing need for help to perform the basic activities of daily living on their own. This often requires them to be in the center, since they have little family support, which is usually one of the most important sources of care and so their impairment is one of the causes of admission to residences [29], or have good family support, but due to work or family issues they cannot take care of a person with impaired physical abilities.

Physical deterioration occurs in many cases, in addition to the worsening of chronic pathologies, due to fear of falling, loss of muscle strength or pathologies that affect mobility. This causes many elderly people residing in nursing homes to decide to use wheelchairs, and this, in turn, further worsens their situation, causing greater difficulty in performing activities of daily living, helping the emergence of pathologies such as depression [30].

The loss of physical capacity in old age includes the appearance of more chronic diseases and the worsening of those that already existed as well as social isolation, and sometimes cognitive and somatic problems. It some cases, this can be explained by laziness in the attempt at self-care, due to family or psychological issues or low education, but it is not related to factors such as sex, having children or not, level of education or age.

Normal cognitive impairment associated with age results in a loss of efficiency in mental processes as people get older. Morphological, metabolic, biochemical, or circulatory changes occur naturally in the brain, which, depending on brain activities and brain plasticity can lead to cognitive alterations. However, the onset of deterioration is not associated with a specific age. There is not an exact year at which an older person initiates this process, since the modifications that initiate these changes fall within normal aging, and the association between cognitive function and age is not linear [31].

For this reason, preventive actions that facilitate the living conditions of institutionalized elderly people and favor active aging and delay dependence are important.

Regarding the limitations presented by the study, we state that as it is an observational study, it would be important to expand the sample to broader contexts given the limited control of the researchers over the possible confounding factors that could interfere with the results of the study.

## 5. Preventive Actions from Music Therapy and Physical Activity

Based on these results and following the recommendations of different European organizations, we describe the possible preventive actions and aspects to be considered for the development of intervention plans and programs in rural residences for the elderly.

The WHO [32] recommendations for adults over 65 years of age are to perform at least 150 min per week of moderate physical activity or 75 min per week of intense physical activity, developed in intervals of at least 10 min.

In the case of not being able to reach the established minimum, it is recommended to stay as physically active as possible.

The following are four preventive actions that can be carried out in geriatric or residential centers:

1. Establish physical and cognitive stimulation programs using music as a motivating element.

Authors such as Paul and Ramsey [33] assert that rhythm produces positive therapeutic effects, and that musical activity improves motor skills impaired by age. Adding music to the activities reduces the dropout rates of the programs [34] and it has also been proven that those activities that combine physical exercise with music are more effective than those with only exercise.

To improve balance and gait through rhythmic stimuli, the recommendation is to use slow rhythms in activities that involve balance and resistance while walking. These changes improve attention and function as an element of distraction to prevent the older adult from being aware of their activity, and thus the exercise acts on all the different segments of the body [33].

It has been shown that the typology of optimal exercises in music therapy should include a combination of exercises such as walking, marching, lateral displacement, and transfers from sitting to standing, with the use of turns that try to imitate daily activities that stimulate the development of balance.

It is necessary that the exercises performed through music therapy stimulate concentration in coordination. This is achieved through a rhythm that provokes a positive response on the auditory stimulation in the older adult. One of the first effects achieved after working with music therapy is the coordination of movements.

2. Design internal and external spaces of the residence in a way that facilitates walking and interpersonal relationships. Pino-Juste et al. [35] recommend locating residences for the elderly in quiet places where they can have natural recreational areas and near population centers so that they do not feel disconnected from their usual environment.
3. To reorganize the catalogue of services provided to the elderly to facilitate their autonomy and independence for as long as possible, creating quality standards that make it possible to evaluate compliance with the measures adopted. To this end, we consider it important to link the services of the geriatric residences themselves to community services, increasing their collaboration, coordination and efficiency and eliminating the duplication of services and bureaucratic procedures [36].
4. Establish collaboration and volunteer programs that allow a more fluid communication between the residences and the citizens. The elderly should feel part of society, not a nuisance parked out of the way because it is no longer useful. The integration of the activities of the residences in the community and vice versa allows greater mutual knowledge and, therefore, that the elderly exercise active citizenship thereby maintaining their vitality and full personal development.

In addition, for the implementation of music therapy and physical exercise programs with a preventive purpose, the following principles should be considered:

1. Early assessment of the individual characteristics of the residents.

In the case of residences located in rural areas, we must consider that the residents are people with a low level of education who have had a high level of physical activity in their working lives, since they have worked mainly in agriculture and in the service sector.

2. Carrying out group interventions whenever possible.

Particularly effective are therapies performed in groups, as they are especially beneficial for increasing socialization, perception, and cognition [37]. It should be noted that the benefits gained by the population will depend largely on the time invested.

3. To use music and physical exercise in a complementary and combined way.

Studies such as Hackney and Bennett [34] that combined rhythm with physical exercise concluded that these should be functional for older adults, but at the same time should allow the development of their full potential so that recovery occurs in a shorter period.

4. Interventions should be extended over time to achieve greater effectiveness.

It is necessary to perform a physiotherapeutic intervention based on music therapy during many sessions in order to determine the long-term results. For the physiotherapeutic exercise routine based on music therapy, it is necessary to implement exercise routines that add to 150 min of exercise per week, combining moderate activities. This type of activity should implement movements that exercise the ranges of motion at least three times per week.

It is recommended to apply this type of intervention on patients at the same level of impairment with exercises focused on their needs, to determine the results at different stages.

5. Take advantage of the natural environment where the centers are located.

The quality of life of an elderly person is closely related to their physical and affective environment, which includes not only adequate health care and the closeness and security offered by family or friends, but also an environment that favors autonomous functioning [38].

6. Perform a comprehensive assessment of the resident upon arrival.

When the resident enters the facility, the team of professionals involved should conduct a comprehensive assessment highlighting physical strength, needs and problems, including information on medical history, cognition, social skills, physical skills, vocational or educational background, emotional state, communication skills, family, and leisure skills [39].

7. Interdisciplinary work of professionals.

Finally, it is recommended that different types of professionals work together, including, in addition to music therapists, physicians, nurses, social workers, physiotherapists, occupational therapists and exercise professionals.

8. To facilitate their continuing education throughout their lives.

We must consider the scarce training of residents in institutionalized centers in rural areas. An increasingly used alternative are universities for the elderly [40]; but also, participation in summer courses, permanent training courses or workshops with different themes. The themes of the courses should consider the interests and motivations of the residents. The cultural options to which they have access should be expanded by increasing the synergies and collaboration between residences and socio-community services.

### Key Point for Occupational Therapy

- Occupational therapists can incorporate physical and cognitive stimulation programs using music as a motivator.
- Occupational therapists verified how it is possible to use music and physical exercise in a complementary and combined way.
- Occupational therapists observed the importance of preventive actions that facilitate the living conditions of institutionalized older people and favor active aging and the delay of dependency.

## Figures and Tables

**Table 1:** Measures of central tendency of the Barthel and Pfeiffer. *n* = 109.

	Pfeiffer	Barthel
Mean	2.88	65.46
Median	2.00	75.00
SD	3.001	31.867
Asymmetry	0.928	−0.713
Kurtosis	−0.082	−0.891
Minimum	0	5
Maximum	10	100
CI 95%	(2.28–3.48)	(64.63–75.97)

**Table 2:** Grouped descriptive of the Barthel and Pfeiffer table. *n* = 109.

Variable	Category	*n*	%
Barthel	Slight	52	47.7
	Moderate	12	11.0
	Independent	18	16.5
	Severely	12	11.0
	Total dependency	15	13.8
Pfeiffer	No assessed	10	9.2
	Normal	51	46.8
	Deficient	35	32.1
	Severely	13	11.9

**Table 3:** Analysis of variance (ANOVA) results for the scales of dependency and memory and orientation in relation to the variable work. *n* = 109.

Variable	Category	*n*	Mean	SD	F	Sig.	Bonferroni
Pfeiffer	Administration	6	2.6	4.33	3.48	0.011	Services-Housewife = 0.001 Services-Farming = 0.008
	Services	28	1.65	2.29			
	Farming	27	4.44	3.34			
	Technician	16	2	2.44			
	Housewife	32	3.14	2.78			
Barthel	Administration	6	52.50	42.02	0.492	0.741	No difference
	Services	8	71.07	29.70			
	Farming	7	63.70	31.85			
	Technician	6	63.44	29.96			
	Housewife	2	65.47	34.08			

**Table 4:** Student’s *t*-test results for the scales of dependency and memory and orientation in relation to the independent variables. *n* = 109.

Variable	Category	*n*	Mean	SD	*t*	Sig.	TE
Pfeiffer	Female	71	3.20	3.069	1.697	0.093	0.191
	Male	28	2.07	2.707			
	Children YES	57	2.42	2.492	−1.788	0.077	−0.174
	Children NO	42	3.50	3.515			
	Primary education	88	2.94	2.945	0.602	0.549	0.089
	High school or higher	11	2.36	3.529			
Barthel	Female	79	62.59	32.244	−1.532	0.128	−0.164
	Male	30	73.00	30.075			
	Children YES	62	66.13	30.453	0.251	0.802	0.024
	Children NO	47	64.57	33.957			
	Primary education	97	65.15	31.229	−0.282	0.778	−0.039
	High school or higher	12	67.92	38.106			

## Data Availability

The data are not publicly available due to confidentiality reasons.
